# Treatment of giant fecalith colonic obstruction in a patient with Duchenne muscular dystrophy using endoscopic injection of hydrogen peroxide: a case report and literature review

**DOI:** 10.3389/fmed.2024.1456246

**Published:** 2024-12-04

**Authors:** Cheng Huang, Zhichao Gao, Yuhang Zhang, Guofeng Li, Lida Ge

**Affiliations:** ^1^Department of Colorectal Surgery, First People’s Hospital of Xiaoshan District, Hangzhou, Zhejiang, China; ^2^Department of Neurosurgery, First People’s Hospital of Xiaoshan District, Hangzhou, Zhejiang, China; ^3^Department of Orthopedics, First People’s Hospital of Xiaoshan District, Hangzhou, Zhejiang, China; ^4^Department of Hepatobiliary Surgery, First People’s Hospital of Xiaoshan District, Hangzhou, Zhejiang, China

**Keywords:** Duchenne muscular dystrophy, fecalith, colonic obstruction, conservative management, gastrointestinal complications

## Abstract

**Introduction:**

Duchenne muscular dystrophy (DMD) is an X-linked recessive genetic disorder primarily affecting cardiac and skeletal muscles, with gastrointestinal obstruction being an infrequent complication.

**Case report:**

We present a 17-year-old boy with DMD (G-to-T transversion at c.4150 in the gene encoding dystrophin protein) who developed severe colonic obstruction due to fecal impaction. Abdominal computed tomography revealed an obstructing fecalith in the left colon (length: 39.5 cm, width: 18.3 cm, height: 12.7 cm). Despite the application of initial conservative measures including fasting, enemas, and fluid resuscitation, the obstruction persisted. Therefore, we performed manual disimpaction and endoscopic injection of hydrogen peroxide, effectively alleviating the obstruction.

**Discussion:**

This case underscores the necessity of devising stage-specific, tailored strategies for the prevention and management of gastrointestinal complications in patients with DMD.

## 1 Introduction

Duchenne muscular dystrophy (DMD), an X-linked recessive disorder, stems from dystrophin gene mutations (1). The lack of dystrophin and its associated proteins weakens connections among sarcolemma, cytoskeleton, and extracellular matrix, causing the sarcolemma to become leaky and highly susceptible to injury (2). DMD is clinically characterized by progressive, symmetrical muscle weakness, typically presenting with pseudohypertrophy of the gastrocnemius muscle (3). It can also impair gastrointestinal smooth muscle function, resulting in dysmotility issues, chronic constipation, and gastroesophageal reflux, but rarely leads to intestinal obstruction (4). An increasing number of studies recommend a multidisciplinary approach to manage DMD, including respiratory care, cardiac care, orthopedic management, and endocrine management. However, there are still gaps in the management of gastrointestinal complications (5). Moreover, managing gastrointestinal complications in patients with DMD poses a unique challenge, owing to the patients’ underlying muscular and cardiac conditions. Herein, we report a case of giant fecal colonic obstruction in a patient with DMD that was successfully alleviated through manual disimpaction and endoscopic injection of hydrogen peroxide.

## 2 Case description

### 2.1 Initial findings

A 17-year-old boy diagnosed with DMD (G-to-T transversion at c.4150 in the gene encoding dystrophin protein) was admitted for abdominal distension persisting for over 3 days. As the disease progressed, he developed gastroesophageal reflux disease (GERD), along with hypokalemia and hypocalcemia. As a result, he has been on long-term oral therapy with prednisone acetate tablets, ranitidine capsules, potassium chloride extended-release tablets, and calcium carbonate D3 tablets. Over the past few years, this patient has experienced progressive muscle atrophy and diminished locomotor ability, now relying primarily on a wheelchair for daily activities. This has resulted in significant limitations in walking and reduced muscle strength in the upper limbs, impacting his ability to perform self-care tasks. Despite normal swallowing function and the ability to eat without difficulty, his eating speed is slow due to muscle weakness, necessitating an appropriate posture to prevent choking. His daily water intake is approximately 1,000 mL, primarily from drinking water and liquid foods. To manage chronic constipation, he has undergone various conservative treatments prior to consultation, including regular use of lactulose and other laxatives, as well as routine abdominal massages and physiotherapy by family members to stimulate bowel movements.

**Figure 1:**
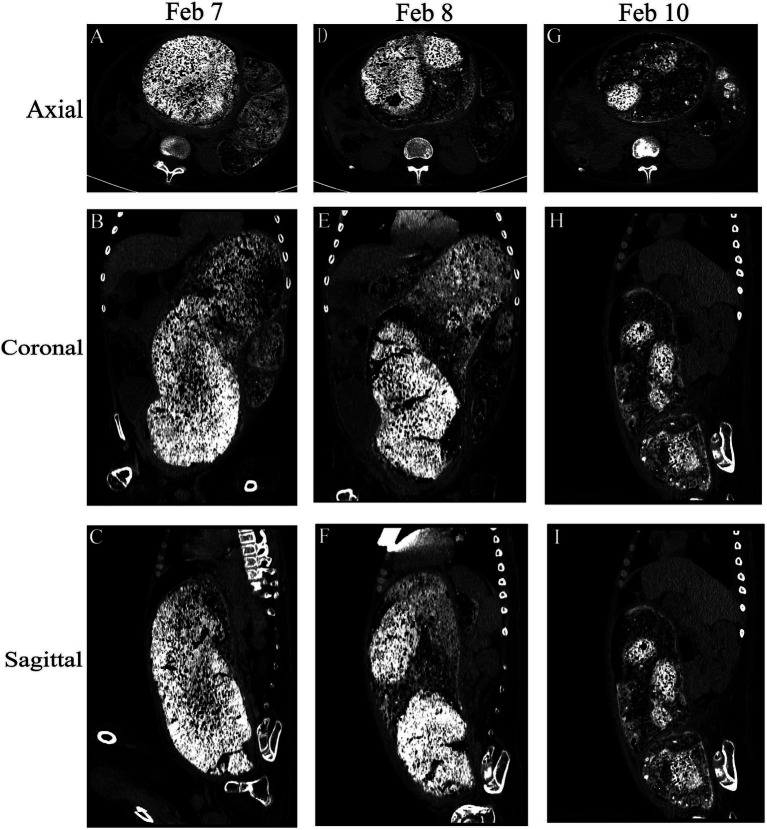
Comparison of the abdominal computed tomography scan before the procedure following multiple enemas. Axial, coronal, and sagittal views were utilized for assessment. **(A–C)** Initial abdominal computed tomography scan revealed a discernible bowel pattern and a notable abdominal mass measuring 39.5 cm × 18.3 cm × 12.7 cm. **(D–F)** and **(G–I)** show the change in fecal characteristics after treatment.

This time, he had not had bowel movements for the past 2 weeks. His vital signs were as follows: temperature 36.8°C, heart rate 96 bpm, respiratory rate 18 breaths/min, and blood pressure 108/78 mmHg. Physical examination revealed abdominal distension due to an appreciable nonspecific intra-abdominal mass, which elicited mild tenderness on palpation without signs of rebound tenderness. The limb, with a muscle strength of grade 2, can move on a flat surface but lacks the strength to overcome gravity and complete the movement. Additionally, the patient exhibited hypokinesia in all limbs, along with bilateral lower limb edema and typical gastrocnemius hypertrophy. A digital rectal examination revealed that the anal sphincter was relaxed, and the rectal lumen was dilated, with a large amount of hard, clustered stool palpated 4 cm from the anal margin. Laboratory tests revealed elevated levels of key biomarkers, including creatine kinase at 2420 (18.0–198.0) U/L and creatine kinase MB isoenzyme at 28.14 (≤0.5) ng/mL. Serum potassium is 3.36 mmol/L, and serum calcium is 2.02 mmol/L. Abdominal computed tomography (CT) indicated dilation of the left colon (width: 18.3 cm), with prominent high-density fecal and gas shadows within the intestinal lumen (Figure 1). The colonic wall exhibited uniform thickening (4–6 mm), and the surrounding organs were visibly compressed owing to the obstruction caused by a large fecalith, with no indication of colonic volvulus.

### 2.2 Treatment and procedure

**Figure 2:**
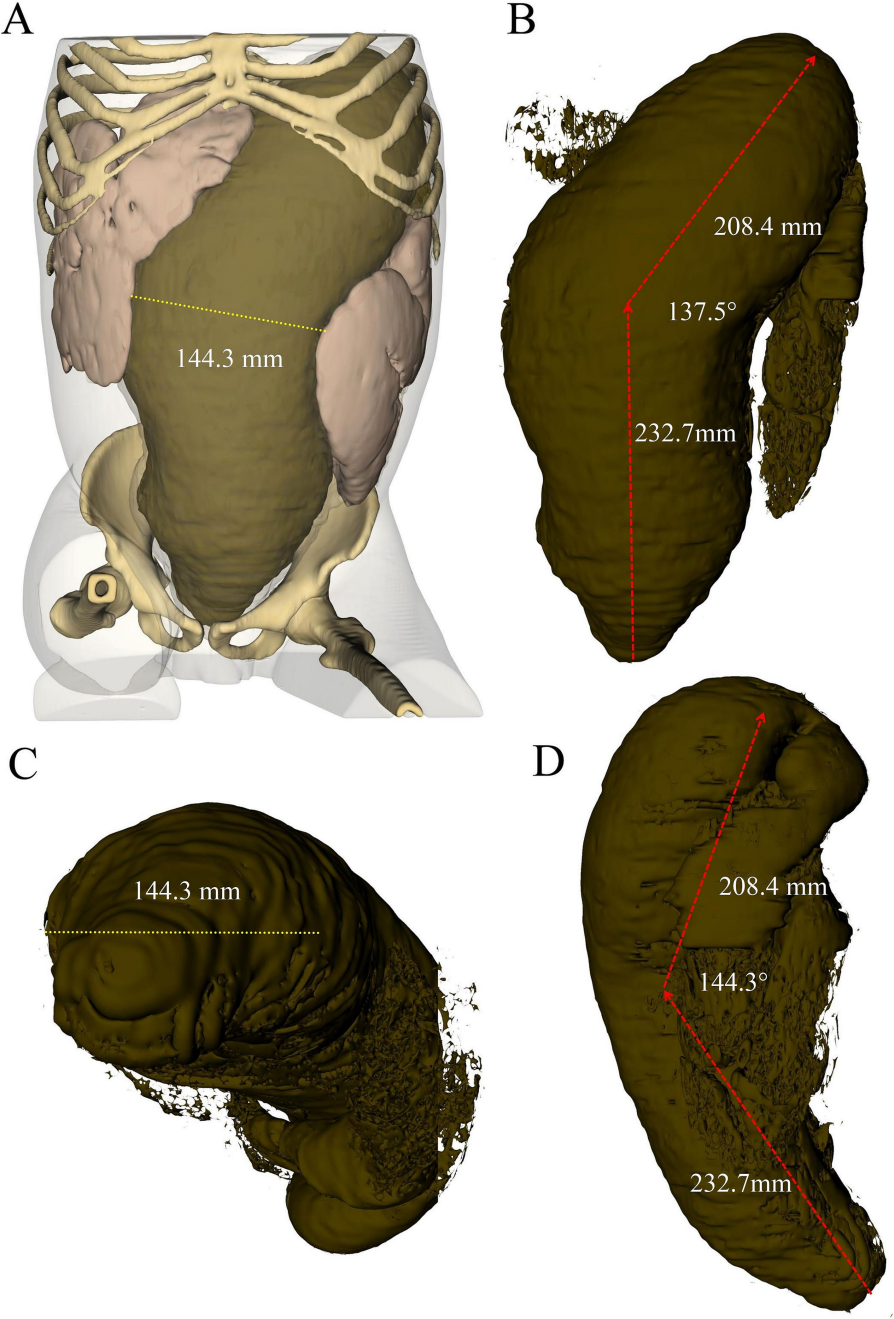
Three-dimensional reconstruction of abdominal CT scan depicting fecalith morphology from different perspectives: **(A)** overall three-dimensional model of the abdomen, **(B)** coronal view, **(C)** axial view, and **(D)** sagittal view.

**Figure 3:**
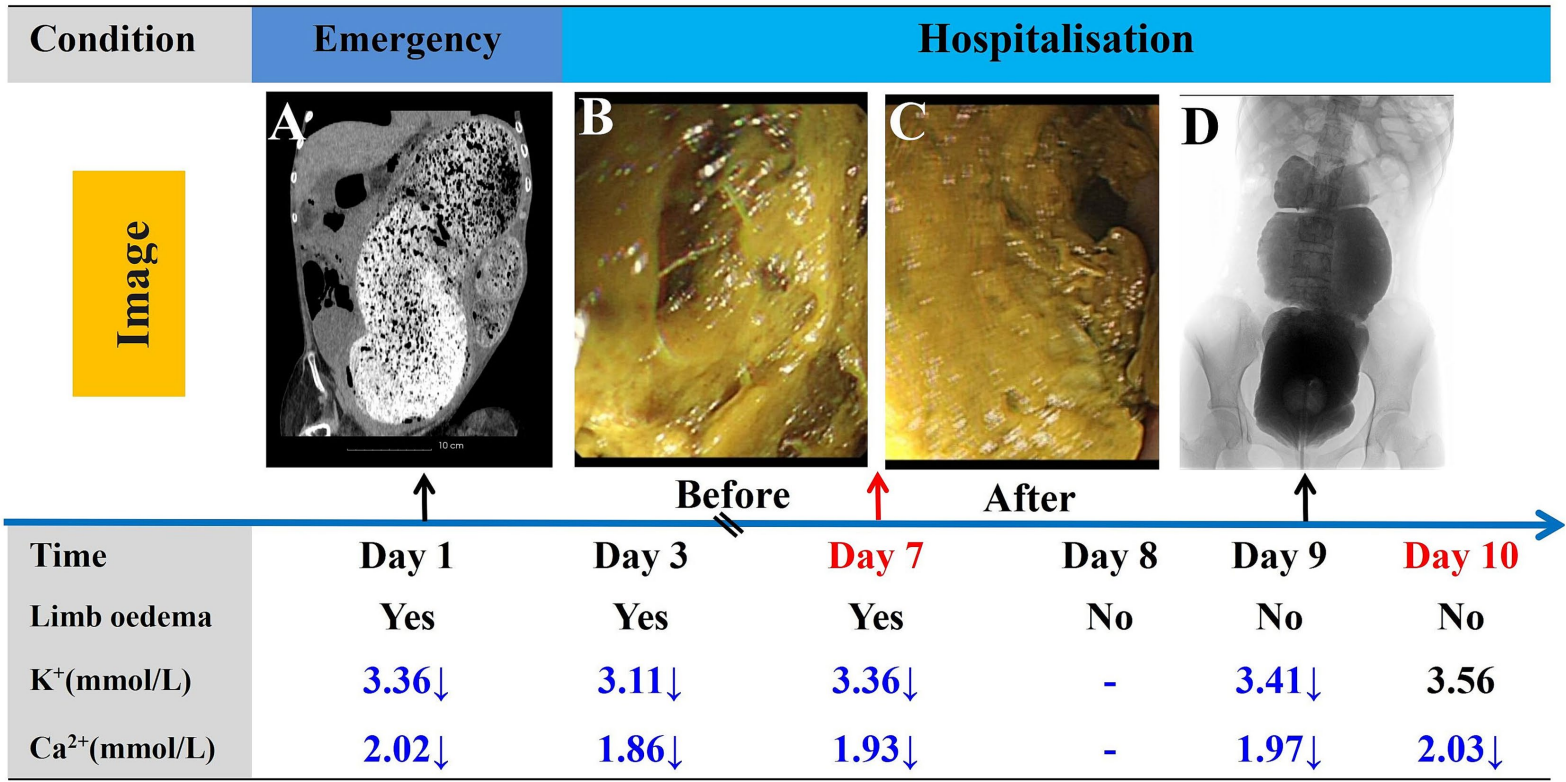
Timeline of the progression and key events during the diagnosis and treatment process. Abdominal CT images taken in the emergency department **(A)**. Changes in intestinal fecal morphology before **(B)** and after **(C)** endoscopic treatment. Barium enema angiography following the resolution of intestinal obstruction **(D)**. Post day 10, regular enemas administered by caretaker.

Upon the patient’s initial visit to the emergency department of our hospital, the patient received a saline enema, leading to the expulsion of a substantial amount of stool and alleviation of abdominal pain. Following this, the patient requested to be discharged home. However, the following day, the patient returned with exacerbated abdominal pain. On admission, the patient was instructed to fast (no gastric tubes were used). Owing to elevated C-reactive protein levels, antimicrobial therapy with intravenous ceftriaxone was initiated. Additional interventions included 10% intravenous potassium chloride for potassium replacement, intravenous omeprazole for gastric mucosal injury prevention, and intravenous calcium gluconate for calcium supplementation. During conservative treatment, we initially attempted endoscopic flushing of the fecal stone and catheter placement for intestinal obstruction; however, owing to the significant size of the fecalith and its hard, stone-like consistency, these measures were ineffective. We performed three-dimensional reconstruction of the abdominal CT scan to reassess fecal morphology and distribution (Figure 2). Subsequently, under direct anoscopic visualization, a portion of the hardened feces was removed using a pair of oval forceps, reaching a depth of 15.0 cm. After establishing the pathway, we injected saline-diluted hydrogen peroxide into the fecalith using a sclerosing needle via sigmoidoscopy until reaching 30 cm into the rectum (Figure 3). We then left the enema catheter in place and administered a 500-mL saline enema, repeating this process three times, which resulted in the dislodgement of a substantial amount of fecal matter. Finally, the enema catheter was secured, and the entire procedure was completed within 1 h.

### 2.3 Results and follow-up

The day after the initial procedure, the patient underwent another enema, leading to the expulsion of a large volume of loose stool in the afternoon, which notably improved the abdominal pain and distension. Subsequently, the patient was discharged from the hospital with a lactulose prescription to aid bowel movement. He has continued to receive two enemas per week, which are administered by his parents. As the patient was predominantly bedridden, regular bowel movements occurred every 2–3 days. Over the 2-year follow-up period, no further instances of intestinal obstruction occurred.

## 3 Discussion

Owing to dystrophin protein production deficiencies caused by DMD, most patients require a wheelchair by 12 years of age (6). Furthermore, premature mortality generally occurs in their third or fourth decade of life owing to respiratory or cardiac complications from diaphragmatic muscle impairment and disease progression without intervention (7). Some patients with DMD may also experience symptoms from gastrointestinal smooth muscle involvement, namely reflux esophagitis, constipation, and life-threatening conditions including intestinal pseudo-obstruction (8). However, fecalith-induced colonic obstruction, resulting from intraluminal hard fecal mass formation, predominantly affects older individuals with chronic constipation (9). The initial clinical presentation is often mild and characterized by progressively worsening abdominal pain and distension with reduced bowel movements. Abdominal CT with coronal and multiplanar reconstruction is commonly used to diagnose colonic obstruction and identify the course of the dilated bowel and exact location of the obstruction (10). If the disease starts acutely, edema of the intestinal wall is also observed on CT, and gas–liquid flatness is observed in the intestinal lumen, often accompanied by intestinal ischemia; if untreated, it can lead to intestinal infarction and perforation (11). In chronic colonic obstruction, compensatory thickening of the colonic wall can occur, even in cases such as ours, where the colon diameter exceeded 18.3 cm without perforation.

Based on previous reports, several potential mechanisms may have contributed to the fecal stone-induced colonic obstruction in our patient. Initially, the absence of dystrophin leads to instability of the Dystrophin-Associated Protein Complex, which in turn results in smooth muscle dysfunction and decreased bowel movements (12). The sigmoid colon, recognized as the most variable segment of the colon, typically assumes an “omega-shaped” configuration, predisposing it to intestinal obstructions (13). Progressive muscle atrophy in the lower limbs limits physical activity, leading to prolonged immobilization (14). With disease advancement, the absence of dystrophin leads to damage in muscle cells, manifesting through oxidative stress, disrupted calcium balance, and compromised sarcolemma integrity. Additionally, this deficiency fosters further cellular impairment, notably affecting the neuromuscular junction and disrupting the differentiation of muscle satellite cells (15). This dynamic intestinal obstruction is characterized by residual fecal matter, creating the nidus for stone formation. Continuous deposition gradually expands the lumen until a large calculus occludes the digestive tract. Additionally, it is important to recognize that low water intake, hypokalemia, hypocalcemia, and cardiac failure are potential causes of intestinal obstruction in patients with DMD and should not be overlooked.

For patients with colonic obstruction, the conservative management strategies typically involve emergency surgery, manual disimpaction, retention enemas, and the placement of an intestinal obstruction catheter (16). We summarize and compare management approaches for severe gastrointestinal complications associated with DMD based on the literature summary in Table 1. In the past, when traditional conservative management proved ineffective for severe colonic fecal impaction leading to intestinal obstruction, surgery often became the optimal choice. However, it must be acknowledged that patients with DMD are at higher risk for anesthesia-related complications, respiratory failure, and heart failure, making surgery not an optimal choice for these patients (17). Some reports have indicated that relieving ileal obstruction can be attempted through colonoscopy (18). Ontanilla Clavijo et al. (19) innovatively used a sclerosing needle to inject Coca-Cola® into the fecaloma, resolving colonic obstruction secondary to sigmoid fecaloma. For gastric outlet obstruction caused by tannin-phytobezoars, administration and endoscopic injection of Coca-Cola® can also effectively alleviate the obstruction (20). Furthermore, Yang et al. (21) successfully treated a giant colonic fecalith causing bowel obstruction by employing endoscopic fenestration combined with catheterization, but this required multiple procedures under colonoscopy. In this case, the patient’s fecalith was so large and hard that initially, we also attempted to soften the feces through colonoscopy, but with limited success. Considering the proximity of the fecalith to the anus, we changed the treatment strategy by first using a proctoscope to create a 15-cm channel under direct visualization, allowing the colonoscope to enter the fecalith. Subsequently, following Ontanilla Clavijo et al.’s approach, we injected saline-diluted hydrogen peroxide into the fecalith using a sclerosing needle. Hydrogen peroxide acts as a leavening agent. As it decomposes, it gradually breaks down the large fecalith into smaller, softer pieces, ultimately enabling the successful relief of the obstruction with a traditional saline enema. In contrast to the previous method, we used a sigmoidoscope for the procedure, as it is more effective in managing obstructions in the rectum and sigmoid colon. It is crucial to thoroughly evaluate the cardiac function of patients with DMD before any procedure to prevent heart failure resulting from invasive operations. Additionally, it is important to acknowledge that hydrogen peroxide releases oxygen, and therefore, procedures like electroresection should not be performed endoscopically.

**Table 1:** Reported cases of Duchenne syndrome with gastrointestinal obstruction.

References	Age (years)	Symptoms	Treatments	Outcome	Invasiveness	Side effects
Bensen et al. (23), USA	19	Severe epigastric pain, gastric dilatation	Ursodiol, intravenous fluids, nasogastric tube, cisapride, ranitidine	Decompression of stomach, improved intake	Non-invasive	Nausea, abdominal discomfort
Jordan-Ely et al. (24), Australia	17	Slow-transit constipation, leg pain	Polyethylene glycol+sodium picosulfate, transcutaneous electrical stimulation (TES)	5–6 L of soft stools, resolved leg pain	Non-invasive	None reported
Dhaliwal et al. (25), USA	21	Abdominal pain, vomiting, chronic constipation	Nasogastric tube, lactulose, enemas	Resolution of gastric distention	Non-invasive	Potential for electrolyte imbalance
Pitrone et al. (26), Italy	22	Severe abdominal pain, chronic constipation	Noninvasive ventilation, subtotal colectomy	Multiple ischemic areas found, surgical intervention	Invasive (surgery)	Surgical risks, recovery time
This article, China, 2023	17	Lower limb edema, constipation	Lactulose, enemas, manual disimpaction, colonoscopy	Relieves intestinal obstruction, lower limb edema	Non-invasive	Mild gastrointestinal discomfort

## 4 Management

A standardized management protocol should be developed for this group of patients to prevent recurrence. This includes regular digital rectal examinations every 3 months to monitor the re-accumulation of hard stool in the rectum and sigmoid colon. Sigmoidoscopy was performed if digital examination findings indicate re-impaction. For patients with DMD experiencing constipation or fecal impaction, daily osmotic laxatives or lactulose may be needed, and retrograde enemas may provide relief in cases of severe impaction. GERD can be managed by reducing gastric acid with histamine 2 receptor blockers, such as ranitidine, or proton pump inhibitors, including lansoprazole and omeprazole (5). Potassium chloride extended-release tablets and calcium carbonate D3 tablets were prescribed for potassium and calcium supplementation, respectively. Dietary approaches to prevent GERD symptoms include eating smaller and more frequent meals and reducing dietary fat intake (22). Compliance was reinforced through counseling on the importance of adherence to the medication regimen. Considering the progressive loss of skeletal muscle function in patients with DMD, aerobic exercises involving the lower limbs are recommended at the early stages of the disease, tailored to the patient’s physical capacity. Regular physical activity aims to enhance gastrointestinal motility through mechanical stimulation. Larger prospective studies are needed to establish evidence-based guidelines for the screening and medical/surgical treatment of this underrecognized complication in the growing DMD population.

## 5 Conclusion

We present a rare case of a giant fecal colonic obstruction in a 17-year-old boy with DMD. Compared to high-risk surgical interventions, our tailored conservative management strategy achieved favorable therapeutic efficacy via meticulous manual extraction of the obstructing fecalith under direct visualization and endoscopic injection of hydrogen peroxide into the fecalith. This case underscores the need for designing individualized treatment plans for patients presenting with intestinal obstruction, particularly for those who are intolerant to surgery.

## Data Availability

The original contributions presented in the study are included in the article/supplementary material, further inquiries can be directed to the corresponding author.
